# Supplementary material for: Long-Acting Recombinant IL-7 (rhIL-7-hyFc) Enhances the Primary and Memory Neoantigen-Specific Immune Response to Breast Cancer Personalized Cancer Vaccines
Source: Cancers (Basel). 2025 Sep 30;17(19):3177. doi: 10.3390/cancers17193177 (PMC12523240; doi:10.3390/cancers17193177)
Supplement: Supplementary file 1 [file cancers-17-03177-s001.zip › cancers-3804246-SI- Figure S1- PCV Kinetics .pdf]

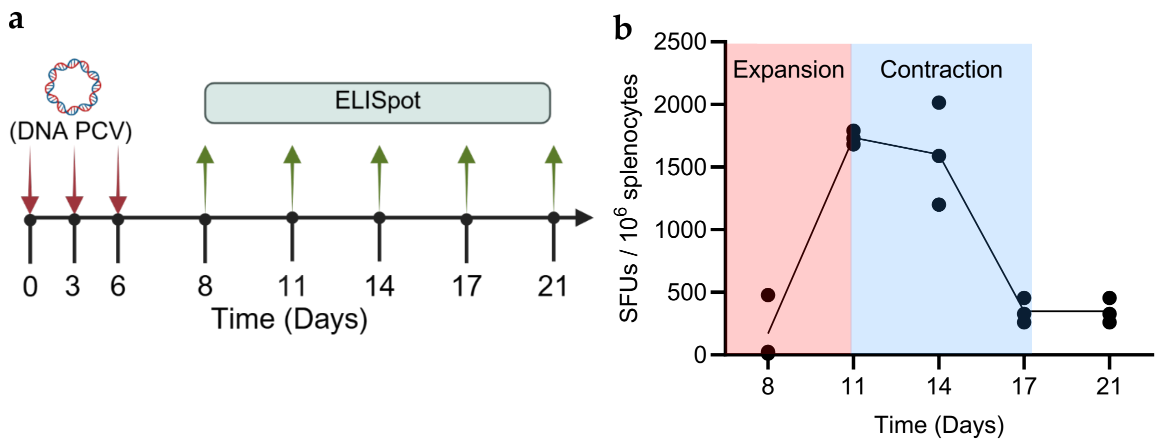

Figure S1. PCV Kinetics**. (a)** Mice (n=15 in total) were vaccinated with three doses of DNA PCV (4 µg per dose) for 3 doses every three days. ELISpot assays were performed (n=3 at each time point) every third day starting two days after completion of vaccination. **(b)** ELISpot assay demonstrates a T cell expansion phase peaking 5 days after completion of vaccination followed by a contraction phase.
