# Supplementary material for: Long-Acting Recombinant IL-7 (rhIL-7-hyFc) Enhances the Primary and Memory Neoantigen-Specific Immune Response to Breast Cancer Personalized Cancer Vaccines
Source: Cancers (Basel). 2025 Sep 30;17(19):3177. doi: 10.3390/cancers17193177 (PMC12523240; doi:10.3390/cancers17193177)
Supplement: Supplementary file 1 [file cancers-17-03177-s001.zip › cancers-3804246-SI- Figure S2.pdf]

a.

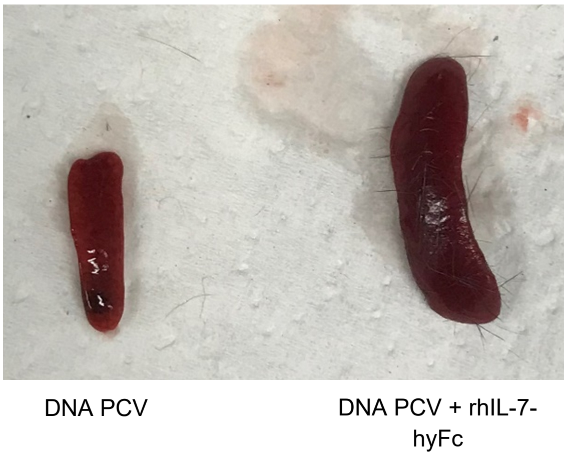

Figure S2. Spleen Size. (**a**) Two representative spleens were harvested from mice (n=2) vaccinated with either DNA PCV alone or DNA PCV + rhIL-7-hyFc. A qualitative difference in size may be observed.
